# Early Intervention in Psychosis in Southwark – Bringing Antipsychotic Prescribing Closer to the Gold Standard

**DOI:** 10.1192/bjo.2024.344

**Published:** 2024-08-01

**Authors:** Samuel Atkinson, Xiaofei Fiona Huang, Jenny Irvine, Yasamine Farahani-Englefield, Margarita Kousteni

**Affiliations:** South London and Maudsley NHS Foundation Trust, London, United Kingdom

## Abstract

**Aims:**

This quality improvement project was conducted in an Early Intervention in Psychosis CMHT (Community Mental Health Team). We aimed to compare prescribing practices to the RCPsych gold standard for treatment of first episode psychosis. Following an initial audit, intervention was completed aiming to improve adherence to these guidelines and thereby the proportion of patients achieving remission.

**Methods:**

An initial audit of the whole CMHT caseload (with exclusions for patients currently admitted to hospital, under the care of a home treatment team or awaiting assessment) was conducted in June 2021. This was completed from information contained in the electronic patient care record. This recorded for each patient details of whether an antipsychotic was recommended, if one was being taken, the dose, if remission was achieved and the number of previously trialled medications. Following this initial audit interventions were completed through designing a one-page flowchart to empower members of the wider multi-disciplinary team (in particular care coordinators) around prompting appropriate medication changes, with an accompanying education session. Following these interventions, a re-audit was completed in March 2023 and the two samples compared through descriptive statistics. In the first audit 269 patients were included (27 exclusions), and in the second 255 (49 exclusions).

**Results:**

The initial pre-intervention audit found that of patients taking medications, 33% (N = 172) hadn't achieved remission. In the follow up audit the proportion of patients taking medication without having achieved remission remained similar at 37% (N = 147). However, the proportion in this group receiving treatment on doses below the licenced maximum improved from 85% (N = 68) to 76% (N = 55). Those on treatment but not in remission who had sufficiently trialled 2 or more antipsychotics (and therefore would meet the criteria for treatment resistance) increased from 50% (N = 52) to 56% (N = 55). The proportion of this treatment-resistant group receiving clozapine remained low, but increased from 3.8% (N = 26) to 9.7% (N = 31).

**Conclusion:**

This project demonstrated modest improvements in prescribing practice, with a small increase in symptomatic patients receiving gold-standard treatment both in terms of numbers of medication trialled and reaching maximum doses. However there remains a significant gap, with a large proportion of symptomatic cases still showing room for medication optimisation. In particular clozapine remains underutilised in this cohort, with only a small minority of patients who would meet the criteria for treatment-resistant psychosis being prescribed it. This leaves room for further interventions to improve prescribing practice.

